# Ten-year adherence to continuous positive airway pressure treatment in patients with moderate-to-severe obstructive sleep apnea

**DOI:** 10.1007/s11325-020-02033-0

**Published:** 2020-02-19

**Authors:** Matsusato Tsuyumu, Tadao Tsurumoto, Jiro Iimura, Tsuneya Nakajima, Hiromi Kojima

**Affiliations:** 1 Department of Otorhinolaryngology, Tokyo Dental College Ichikawa General Hospital, Chiba, Japan; 2 Department of Otorhinolaryngology, Jikei University School of Medicine, 3-25-8 Nishi-shimbashi, Minato-ku, Tokyo, 105-8461 Japan

**Keywords:** Obstructive sleep apnea, Polysomnography, Body mass index, First-month utilization rate, Long-term continuation, Continuous positive airway pressure

## Abstract

**Purpose:**

This study aimed to evaluate the 10-year adherence to and identify the predictors of dropout from continuous positive airway pressure (CPAP) treatment for patients with moderate-to-severe obstructive sleep apnea (OSA).

**Methods:**

We retrospectively analyzed the continuity, dropout, or other behaviors of 181 patients who initiated CPAP treatment at the Tokyo Dental College Ichikawa General Hospital from January 2003 to June 2005.

**Results:**

Among a total of 181 patients, 56 (30.9%) dropped out of the treatment. Among the 125 patients who did not dropout, 54 continued CPAP treatment for > 10 years, 16 completed the treatment with OSA improvement, and 7 could not complete the treatment owing to unavoidable reasons such as death, dementia, hospitalization for serious illness, or migration to other countries. Further, 47 patients moved to another facility, whereas 1 patient purchased a CPAP device and stopped visiting our facility. Among the 56 patients who dropped out, approximately 50% of the patients dropped out within a year, and all dropped out within 76 months. Comparing demographics, OSA parameters, and CPAP parameters between the patients who did and did not drop out of the treatment, Cox regression analysis indicated that body mass index (BMI) and the first-month utilization rate were clinical variables that were independently associated with discontinuation of CPAP treatment.

**Conclusion:**

The results of this study show that BMI and the first-month utilization rate of CPAP treatment are the predictors of the long-term adherence to this treatment.

**Electronic supplementary material:**

The online version of this article (10.1007/s11325-020-02033-0) contains supplementary material, which is available to authorized users.

## Introduction

Obstructive sleep apnea (OSA) is a very common respiratory disorder that is estimated to affect 936 million individuals aged 30–69 of the worldwide population [1]. OSA leads to an increase in metabolic diseases, neurological disorders, cardiovascular diseases, and mortality rates [2–4].

Continuous positive airway pressure (CPAP) improves subjective symptoms, such as daytime sleepiness, cognitive performance, blood pressure, and quality of life [5–9]. Long-term prognosis is affected by adherence to CPAP treatment rather than OSA severity [10].

CPAP treatment’s one- [11, 12], 2- [13], and 3-year continuation rates [14] have been reported; however, CPAP treatment should be continued for longer than 3 years. Although long-term continuation studies have been conducted [15–23], in many studies, it is not clear as to how many years are considered long term. Although we considered that “10 years” was a relatively long period of time, few reports analyzed in detail whether patients with OSA continued CPAP treatment, dropped out of CPAP, or followed other courses for a long period of 10 years after CPAP was initiated. The purpose of this study was to retrospectively examine the progress 10 years after the start of CPAP and to investigate factors related to its continuation/omission, while targeting patients with OSA who started CPAP more than 10 years ago.

## Methods

### Patients

Overall, 280 patients suspected of OSA underwent polysomnography (PSG) with hospitalization at the Tokyo Dental College Ichikawa General Hospital for the first time from January 2003 to June 2005. In Japan, CPAP treatment for patients with OSA with an apnea–hypopnea index (AHI) of ≥ 20/h is covered by government health insurance. As per the results of PSG, 196 patients had an AHI of ≥ 20/h. In this study, we provided CPAP treatment to 181 patients. In accordance with the Japanese health care system, all patients using CPAP treatment were required to visit medical institutions every month to report their usage.

All PSG and CPAP titrations in the facility were conducted using the Alice4 system (Philips, Respironics Inc., Murrysville, PA, USA). All PSG were manually analyzed according to the standard criteria [24]. Apnea was defined as a lack of airflow that lasted for > 10 s. Hypopnea was defined as a decrease in the airflow by approximately 50% for at least 10 s or a decrease of 3% in oxygen saturation [25]. Moreover, 181 patients underwent CPAP titration on the second night of hospitalization using an automatic CPAP device under the supervision of skilled staff. When a significant increase in pressure was observed, the pressure was adjusted by the staff. A pressure range was obtained to normalize the breathing pattern and minimize arousals. After the titration test, automatic CPAP treatment was provided to the patient.

### Procedures

#### Investigation of CPAP continuation

CPAP application (the number of days that CPAP was used by patients with OSA at their home in 1 month) was confirmed every month. A memory card in the CPAP device recorded the days on which CPAP was used and not used. We recorded the number of days of CPAP use for each patient. Patients who reported difficulty in CPAP application owing to nasal closure symptoms were treated with a nasal steroid to be used topically or an oral anti-allergy agent. For patients who complained of discomfort during use, the pressure was adjusted and the mask type was changed. For patients who still wished to drop out of CPAP treatment, we also recommended other methods such as oral appliances or surgery. On the contrary, among the patients who continued CPAP for improving OSA, those who exhibited decreased obesity owing to diet and exercise therapy or underwent surgery for OSA were examined by conducting PSG again. Patients with an AHI decreased to < 20/h were withdrawn from CPAP. Patients with AHI of ≥ 5/h but < 20/h discontinued CPAP according to the Japanese health care system and switched to treatment with the oral appliance. Patients with AHI of < 5/h were considered to have sufficiently improved OSA and discontinued treatment.

CPAP treatment was terminated following the death of patients or following difficulty of use due to dementia. Some patients faced difficulties in visiting our facility owing to serious illnesses requiring long-term hospitalization. In addition, some patients had to stop using CPAP under the Japanese health care system due to overseas migration.

Patients who were no longer able to visit our facility owing to relocation within Japan decided to continue CPAP treatment under the Japanese health care system and visit hospitals located near their current location. Therefore, the subsequent use of CPAP treatment is unknown.

Adherence to CPAP 10 years after its initiation was defined and roughly classified into the following two categories.

Patients refusing CPAP: Patients who refused and dropped out of CPAP treatment. These patients underwent CPAP treatment and were instructed to use it, but later, they did not wish to continue CPAP or did not use CPAP at all.

Patients accepting CPAP: Patients who used CPAP treatment and did not dropout of CPAP treatment under our observation. This group was further classified as follows:

Continuation: Patients who continued CPAP treatment for > 10 years. The following two points were considered necessary for continuing CPAP treatment: (1) those who wished to continue using CPAP treatment and (2) those who continued to visit our facility once a month.

Withdrawal: AHI decreased owing to events such as weight loss and/or surgery, and CPAP was determined to be unnecessary. Depending on the AHI, some of the patients switched to treatment with the oral appliance and others completed treatment with OSA.

Termination: CPAP was terminated owing to unrelated complications or inevitable reasons, such as death, advanced dementia, serious illness, or emigration.

Transfer to another facility: Patients decided to continue CPAP at another facility instead of ours.

CPAP self-management: One patient refused to visit our medical facility and purchased a CPAP device for OSA self-management.

#### Period until the dropout of CPAP treatment

Regarding patients refusing CPAP, the period from the initiation of CPAP treatment to its discontinuation was summarized. The continuation rate of 10 years was evaluated according to the Kaplan–Meier survival analyses, and the number of months required for the continuation rate to reach the plateau was estimated.

#### Factors related to the dropout of CPAP treatment

Each patient’s clinical background, OSA parameters obtained from PSG, and parameters obtained from CPAP titration were evaluated to determine the relationship between adherence-related events and the following clinical background parameters: sex, age, body mass index (BMI), lifestyle (smoking and drinking habits), Epworth Sleepiness Scale (ESS) before the initiation of CPAP, nasal airflow resistance before the initiation of CPAP, and the first-month utilization rate, which is the ratio (percentage) of the days using CPAP to the days from the day of initiation of CPAP to the outpatient visit date 1 month later. Furthermore, we investigated AHI, apnea index, hypopnea index, minimum oxygen saturation (SaO_2_), and time spent with SaO_2_ < 90% measured using PSG and CPAP titration.

A univariate Cox regression analysis was performed to investigate the association between the dropout of CPAP treatment and the clinical background of the patients, parameters obtained by PSG or CPAP titration, and factors that were added after CPAP was initiated.

A Cox multivariate analysis was performed for each factor that was found to affect dropout from CPAP treatment with *p* < 0.1 in univariate analysis. AHI is an important factor that indicates the severity of OSA and was thus entered into a multivariate analysis regardless of the results of univariate analysis. The assumption of proportional hazards was graphically verified using a log–log survival curve. The results of Cox multivariate analysis were expressed as the risk ratio with 95% confidence interval, and *p* < 0.05 was considered statistically significant. This research was approved by the Tokyo Dental University Ethics Committee.

## Results

The baseline characteristics (sex, age, BMI, smoking and alcoholic status, ESS, and nasal airflow resistance) of the 181 patients included in the study have been summarized in Table 1.

**Table 1:** Baseline characteristics of the 181 patients in the study

Sex (male/female)^a^	163/18 (90.1/9.9)
Age (years)^b^	48 (37–60)
BMI (kg/m^2^)^b^	27.4 (24.6–30.6)
Smoke (yes/no)^a^	66/115 (36.5/63.5)
Drink alcohol (yes/no)^a^	105/76 (58.0/42.0)
ESS^b^	8 (5–10)
Nasal airflow resistance (Pa/cm^3^/s)^b,c^	0.571 (0.364–1.180)

^a^Sex, smoking, and drinking are indicated by the number of cases (%)

^b^Age, BMI, ESS, and nasal airflow resistance are indicated by the median (interquartile range)

^c^Nasal airflow resistance was measured as ΔP 100 Pa during spontaneous nasal inspiration breathing using anterior rhinomanometry with the patient in a supine position

*BMI* body mass index, *ESS* Epworth Sleepiness Scale

### A 10-year course following the initiation of CPAP treatment in patients

**Fig. 1:**
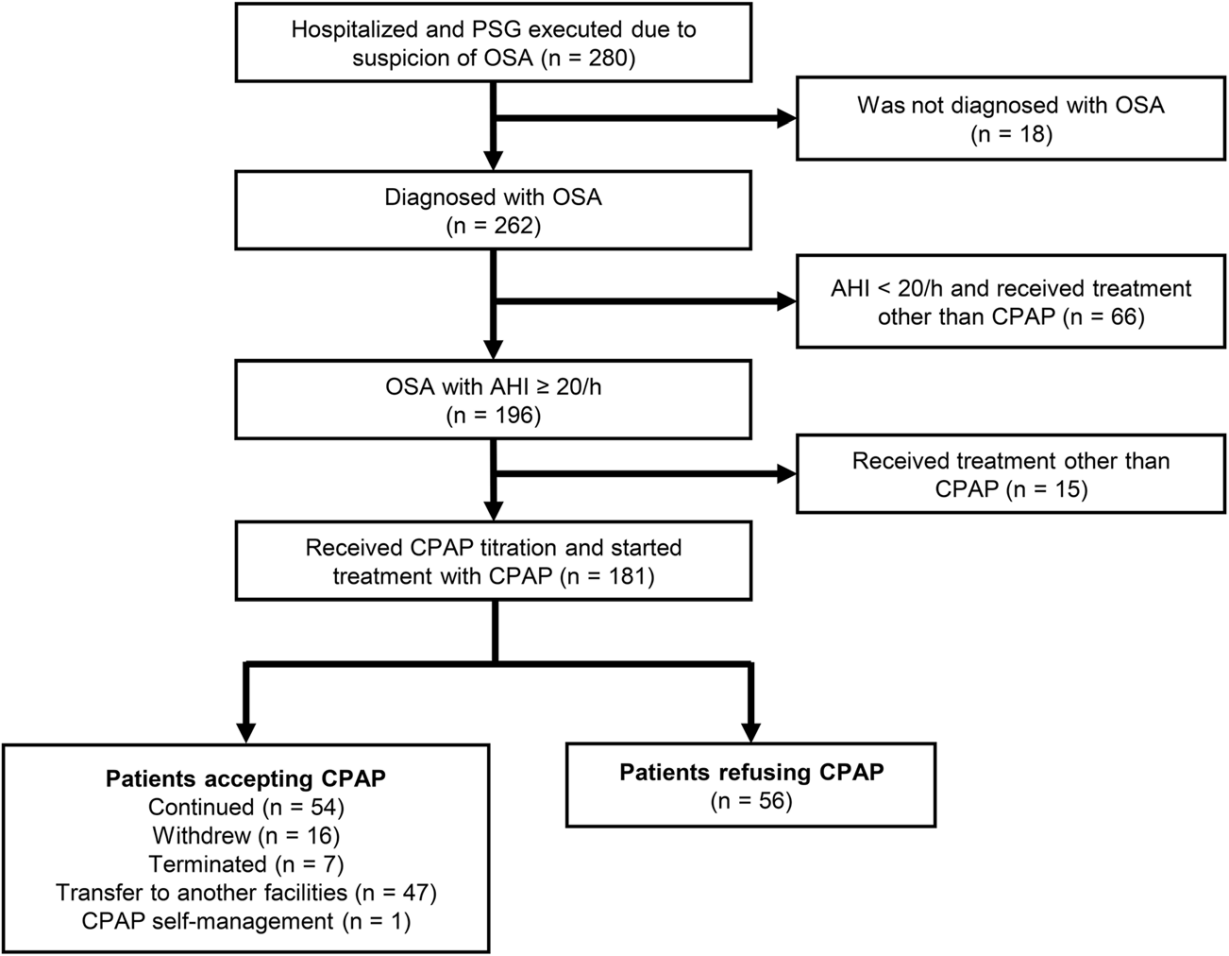
Flow diagram of study participants. OSA, obstructive sleep apnea; CPAP continuous positive airway pressure; AHI apnea–hypopnea index; PSG polysomnography

Overall, 56 patients (30.9%) refused CPAP treatment and dropped out. Among the remaining 125 patients (69.1%) who did not dropout of CPAP treatment, 54 continued CPAP treatment for > 10 years, 16 completed the treatment with OSA improvement, and 7 could not complete the treatment owing to unavoidable reasons such as death, dementia, hospitalization for serious illness, or migration to other countries. Further, 47 patients moved to another facility, and 1 purchased a CPAP device and stopped visiting our facility (Fig. 1).

### Period until the dropout of CPAP treatment

**Fig. 2:**
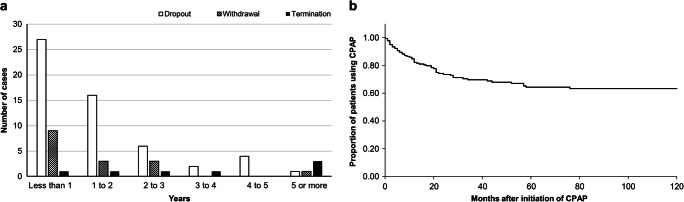
**a** Number of years/patients until discontinuation in cases of patients refusing CPAP. **b** Kaplan–Meier curve presenting the proportion of patients on CPAP therapy versus time

The median duration from the initiation of CPAP treatment to its dropout was 12 (interquartile range (IQR) 4.5–21.5) months. Approximately half of the dropout patients discontinued CPAP treatment within 12 months of its initiation. The median duration from the initiation of CPAP treatment to its withdrawal with was 11.5 (IQR 6.5–14.5) months and to its termination with was 37 (IQR 18.5–87.8) months. The longest duration until dropout was 76 months (Fig. 2a). Eight (14.3%) out of 56 patients underwent other treatments, such as that involving oral appliances or surgery, whereas the remaining patients refused treatment for OSA. To calculate the 10-year retention rate, withdrawal, termination, transfer to another facility, and CPAP self-management groups were considered “censored” variables. It was estimated that 63.3% of the patients continued CPAP for 10 years according to the Kaplan–Meier method and that the proportion of the patients who continued CPAP treatment reached a plateau in nearly 76 months (Fig. 2b).

### Factors related to the dropout of CPAP treatment

Based on the results of univariate analysis, the BMI of patients accepting CPAP was significantly higher than that of patients refusing CPAP. The first-month utilization rate after the initiation of CPAP treatment was significantly higher for patients accepting CPAP than for patients refusing CPAP. There was no statistical difference between sex, age, smoking habits, drinking habits, ESS, or nasal airflow resistance between the two groups (Table 2).

**Table 2:** Univariate analysis: Variables affecting dropout of CPAP (clinical background)

	Patients refusing CPAP (*n* = 56)	Patients accepting CPAP (*n* = 125)	Hazard ratio (95% CI)	*P* value
Sex: male/female^a^	50/6 (89.3/10.7)	113/12 (90.4/9.6)	0.87 (0.37–2.02)	0.746
Age (years)^b^	49 (36–63)	49.5 (39–60)	0.99 (0.97–l.01)	0.214
BMI (kg/m^2^) ^b^	26.0 (23.9–28.6)	27.9 (24.8–31.0)	0.92 (0.86–0.97)	0.005
Smoke^a^ (yes/no) ^a^	22/34 (39.3/60.7)	43/82 (34.4/65.6)	1.40 (0.82–2.40)	0.216
Habit of drinking alcohol (yes/no) ^a^	27/29 (48.2/51.8)	73/52 (58.4/41.6)	1.06 (0.63–1.81)	0.816
ESS ^b^	8 (5–10)	8 (5–10)	1.00 (0.94–1.06)	0.980
Nasal airflow resistance (Pa/cm^3^/s) ^b^	2.1 (0.3–1.2)	0.57 (0.4–1.00)	1.04 (0.97–1.10)	0.282
First-month utilization rate (%)^b c^	70.8 (51.1–95.1)	92.8 (74.3–100)	0.98 (0.97–0.98)	< 0.001

^a^Sex, smoking, and drinking are indicated by the number of cases (%)

^b^Age, BMI, ESS, and nasal airflow resistance are indicated by the median (interquartile range)

^c^Nasal airflow resistance was measured at ΔP 100 Pa with anterior rhinomanometry

BMI body mass index, *CPAP* continuous positive airway pressure, *ESS* Epworth Sleepiness Scale

A low minimum SaO_2_ value measured by PSG was associated with CPAP treatment continuation, as observed in the results of PSG (*p* < 0.1) (Table 3).

**Table 3:** Univariate analysis: Variables affecting dropout of CPAP (OSA parameters)

PSG	Patients refusing CPAP (*n* = 56)	Patients accepting CPAP (*n* = 125)	Hazard ratio (95% CI)	*P* value
AHI (/h)	47.7 (36.1–72.1)	48.3 (32.3–76.8)	1.00 (0.99–1.01)	0.599
AI (/h)	15.8 (7.9–45.2)	19.7 (7.8–57.0)	1.00 (0.99–1.00)	0.339
HI (/h)	23.2 (16.7–32.3)	20.3 (11.8–28.3)	1.01 (0.99–1.03)	0.215
Minimum SaO_2_ (%)	80 (72–84)	77 (69–82)	1.03 (1.00–1.06)	0.085
Time (SaO_2_ < 90%) (min)	16.0 (8.3–63.3)	28.0 (8.6–75.4)	1.00 (0.99–1.00)	0.200

The value of each item is indicated by the median value (interquartile range)

*AHI* Apnea–hypopnea Index, *AI* Apnea Index, *HI* Hypopnea Index, *CPAP* continuous positive airway pressure, *PSG* polysomnography, *OSA* obstructive sleep apnea, *Time (SaO2 < 90%)* time spent with SaO2 < 90%

There was no statistical difference between the two groups with regard to OSA parameters obtained by CPAP titration (Table 4).

**Table 4:** Univariate analysis: Variables affecting dropout of CPAP (CPAP titration)

CPAP titration	Patients refusing CPAP (*n* = 56)	Patients accepting CPAP (*n* = 125)	Hazard ratio (95% CI)	*P* value
AHI (/h)	6.2 (3.8–12.8)	7.2 (3.9–9.9)	1.00(0.97–1.04)	0.954
AI (/h)	0.6 (0.2–1.7)	0.5 (0–1.2)	1.03(0.96–1.11)	0.394
HI (/h)	5.3 (3.3–8.4)	5.3 (3.4–8.7)	0.99(0.95–1.04)	0.746
Minimum SaO_2_ (%)	90 (88–93)	90 (87–92)	1.03(0.97–1.09)	0.403
Time (SaO_2_ < 90%) (min)	0 (0–1.1)	0 (0–1.0)	0.96(0.90–1.04)	0.374

The value of each item is indicated by the median value (interquartile range)

*AHI* Apnea–hypopnea Index, *AI* Apnea Index, *HI* Hypopnea Index, *CPAP* continuous positive airway pressure, *PSG* polysomnography, *OSA* obstructive sleep apnea, *Time (SaO2 < 90%)* time spent with SaO2 of < 90%

Factors with *p* < 0.1 in univariate analysis and AHI were included in multivariate analysis. The Cox multivariate analysis was performed, and it was indicated that BMI (*p* < 0.001) and the first-month utilization rate (*p* < 0.001) were significantly associated with CPAP continuation for over 10 years (Table 5).

**Table 5:** Multivariate analysis: variables affecting dropout of CPAP

	Hazard ratio (95% CI)	*P* value
First-month utilization rate (%)	0.97(0.96–0.98)	< 0.001
BMI (kg/m^2^)	0.89(0.84–0.96)	< 0.001

*BMI* body mass index

## Discussion

This study evaluated factors affecting the continuation and dropout of CPAP treatment 10 years after its initiation in patients with OSA. Less than half of the patients (30.9%) dropped out of the treatment. The withdrawal, termination, transfer to another facility, and CPAP self-management groups were considered censored cases, and the 10-year continuation rate was estimated to be 63.3% as per the Kaplan–Meier method. Patients refusing CPAP treatment discontinued it within 76 months from its initiation. Based on the results of multivariate analysis, BMI and first-month utilization rate immediately after the initiation of CPAP treatment were associated with the continuation of CPAP treatment.

In previous studies, the 10-year continuation rate was estimated to be 64.1% [16], 70% [22], or 79.9% [23]. Compared with the previous predictions, the number of patients who dropped out was high and the number of those who continued CPAP treatment was low. The reason for this difference may be the differences in approaches across hospitals and countries. The number of patients who fail to continue treatment after being diagnosed with OSA may be higher than that predicted in the past.

In a previous study, no patient dropped out of CPAP treatment after 48 months from its initiation [19]. In the present study, the dropout was confirmed within 76 months. It should be recognized that patients with OSA might discontinue CPAP treatment until 76 months.

Some studies suggest that BMI has no effect on CPAP treatment continuation [11, 12, 23]. However, others suggest that BMI does effect CPAP treatment continuation, and the findings of the present study are consistent with the results of these studies [13, 21]. One study found that patients with drowsiness had high BMI and proposed that the decrease in drowsiness caused by the application of CPAP treatment would motivate patients to continue CPAP application [13]. Patients with a high BMI may experience the benefits of CPAP treatment more easily. One study comparing obese patients with OSA with non-obese patients with OSA shows that OSA non-obese patients have a lower arousal threshold for airway stenosis, which may limit CPAP resistance and contribute to reduced CPAP compliance [26]. It should be recognized that it may be difficult to treat OSA with CPAP in patients with low BMI.

The utilization of CPAP treatment, i.e., CPAP usage time per night, within 1–3 months after its initiation was associated with long-term CPAP treatment continuation [13, 19]. Although the present study evaluated the usage in days per month (patients with OSA did not use CPAP treatment on a daily basis, except some patients who used it daily), not the usage time per night, the results were similar to those of previous studies. Some patients may feel comfortable with CPAP treatment and others may not [27–29]. For patients who feel uncomfortable with CPAP treatment since its initiation, it is possible that there are fewer days of CPAP application within the first month, and as a result, these patients may discontinue the treatment. In previous studies, the CPAP application pattern was determined within the first few weeks of treatment initiation [13, 23]. This means that patients who apply CPAP less frequently during its initiation will find it difficult to increase the frequency of use later. Our methods to address patients who feel uncomfortable with CPAP include pressure adjustment, nasal closure measures, and/or mask replacement. These interventions should be implemented as early as possible after the initiation of CPAP treatment.

### Limitations

Our study has some limitations because of its single-facility, retrospective design and relatively small sample size. First, we did not develop strict classification criteria for continuation/dropout based on days of CPAP use. Regarding patients with OSA who were experiencing CPAP-related discomfort, we provided support to improve their CPAP-related experience on days of use. We recommended patients with OSA to use CPAP for at least 4 h for 70% of the days in a month. However, some patients who were classified as patients accepting CPAP failed to exceed this criterion, either temporarily or continuously. We might have to consider whether they were fit to be classified as patients accepting CPAP. Second, mental and psychological factors may affect final results; however, these factors were not considered in this study. Third, most patients in this study were males; therefore, we cannot exclude the possibility of sex differences. Fourth, as a result of using the Japanese health care system, we only provided CPAP to patients with OSA with an AHI of > 20; therefore, CPAP adherence among patients with mild OSA remains unknown. A prospective, multi-institutional study involving patients with mild OSA and a higher proportion of women is required.

Finally, the continuation rate for the past 10 years may not coincide with that in the upcoming 10 years following a recent initiation of CPAP treatment. We believe that the current CPAP machine and mask may be better and easier to use than it was 10 years ago. The continuation rate 10 years from now may be better than that reported in this study.

## Conclusion

In this study, the 10-year continuation or discontinuation rate following the initiation of CPAP treatment was confirmed in patients with OSA. Patient’s BMI and utilization rate within approximately 1 month of the initiation of CPAP treatment are the predictors of long-term CPAP treatment utilization.

## Electronic supplementary material

ESM 1 (DOCX 48 kb)
